# The Medical Society of the State of New York

**Published:** 1889-03

**Authors:** 


### *THE MEDICAL SOCIETY OF THE STATE OF NEW YORK.*

The eighty-third annual meeting of this time-honored Society, held in Albany February 5th, 6th and 7th, will ever be a memorable one, whether considered from the standpoint of the scientific work that it accomplished, or whether from that other but quite important point of view as a reunion of medical men from different portions of the Empire State to renew acquaintance, and burnish the links in the chain of a common friendship that should ever bind men together, who work together in the cause of humanity and science.

The attendance, both as to quantity and quality, was unusually good, and the volume of work accomplished was large. This was to be expected, when it is considered that the President, Dr. S. B. Ward, came into office with a large experience in the management of the affairs of the Society, as well as an extended personal acquaintance with medical men in every portion of the State.

The President's inaugural address, though brief, was commendable therefor, in that it presented succinctly the special points which it is the privilege of the President to bring before the Society for its action. The Society then went directly to its work, and seemingly never stopped, except for the usual recesses, until it concluded its labors.

Two or three points of departure from the usual custom served to add the charm of novelty to the whole proceedings. President Ward's reception on Tuesday evening, after the scientific session, to which the entire Society was invited, besides many others—its friends, and friends of the President—infused new interest and added gracefulness to the occasion.

The second notable departure from the usual custom was the delivery of the President's Annual Address at the opening of the afternoon session on the second day in the hall of the scientific meeting, instead of in the Assembly Chamber in the evening, as has been the fashion for so many years. This can be commended from all points of view as a step in advance.

President Ward's address dealt mainly with the question of medical experts in the courts, described the difficulties of the present system in a most original and striking manner, and, finally, offered the most practical solution thereof that, to our mind, has yet been done, in the following closing paragraphs:

> "In most cases where medical expect evidence is required, at least two physicians are called to the stand, and in many cases a half-dozen. The remedy which we would suggest would be, that under such circumstances a board of three experts should be appointed by the court: one on the suggestion of the counsel for the defense, one nominated by the counsel for the prosecution, and a third by the court itself; that these experts should be paid by the court, and the charge be divided equally between the two sides; that to this board of experts should be submitted in writing the questions involving medical matters; that the answers should be submitted in writing and sworn to, and that medical witnesses should not be required to go upon the stand. In the event of the failure of the board to entirely agree, a minority report might be admitted, and if each side desired to be represented by two or three experts instead of a single one, there would be no exception to such a course.
>
> "The adoption of this course would certainly result in obtaining from medical experts opinions free from the bias which arises from the expectation of pecuniary reward from either side, the unseemly antagonisms between the expert on the stand and the cross-examining counsel would be avoided, and the ends of justice be more speedily and surely attained."

Dr. Wm. Manlius Smith, who for twelve years so honorably conducted the office of Secretary, declined to have his name presented for renomination. This imposed upon the Nominating Committee the duty of selecting his successor, which is no light responsibility in a society like this. The success of any society depends largely upon the ability and the executive capacity of its Secretary; and this is true in a most emphatic way of the Medical Society of the State of New York. There are important questions constantly arising of a legislative, judicial, and scientific nature that must keep the Secretary constantly on the alert to meet emergencies that may arise. It is believed that in the selection of Dr. F. C. Curtis, of Albany, the Society has done itself credit, by choosing a man well equipped for the office in question.

The following is a list of the principal officers chosen: *President,* Daniel Lewis, of New York; *Vice-President,* Alfred Mercer, of Syracuse; *Secretary,* F. C. Curtis, of Albany; *Treasurer,* C. H. Porter, of Albany. *Committee of Arrangements:* Samuel B. Ward, of Albany; Edward L. Partridge, of New York, and C. S. Merrill, of Albany. *Committee on By-Laws:* F. A. Castle, of New York; A. R. Simmons, of Utica, and F. C. Curtis, of Albany. *Committee on Hygiene:* E. V. Stoddard, of Rochester; A. N. Bell, of Brooklyn; Wm. B. Brown, of Bath; J. P. Creveling, of Auburn; Wm. H. Bailey, of Albany; Wm. C. Bailey, of Albion; E. F. Brush, of Mt. Vernon. *Committee on Legislation:* D. B. St. John Roosa, of New York; Burke Pillsbury, of Middletown; Maurice J. Lewi, of Albany. *Committee on Ethics:* A. Jacobi, of New York; Arthur Mathewson, of Brooklyn; H. R. Hopkins, of Buffalo. *Committee on Prize Essays:* Geo. F. Shrady and F. P. Foster, of New York; Eugene Beach, of Gloversville. *Committee on Publication:* F. C. Curtis, of Albany; John O. Roe, of Rochester; O. B. Douglas, of New York; C. H. Porter, of Albany.

*Honorary Members:* Howard Marsh, of London; Francis Bacon, of New Haven; Reginald Harrison, of Liverpool; Morell Mackenzie, of London; William Pepper, of Philadelphia; Max Schede, of Hamburg. *Eligible Honorary Members:* William H. Taylor, of Cincinnati; Joseph Price, of Philadelphia; George J. Engelmann, of St. Louis; R. J. Levis, of Philadelphia; Lennox Browne, of London; James Nevins Hyde, of Chicago; E. E. Montgomery, of Philadelphia.

The President-elect, Dr. Daniel Lewis, of New York, comes into possession of an unsought office, fully equipped to discharge the duties thereof in a most satisfactory manner. He has for years been largely interested in, and intimately associated with, the management of the affairs of the Society, and it is doubtful if any member is more familiar with its minutest detail work. His election will give undoubted satisfaction to every portion of the State.

Finally, a most important step was taken to amend the by-laws, so that a two-thirds majority of the Nominating Committee may make the nomination for President, Vice-President, Secretary and Treasurer, instead of this requiring a unanimous vote, as heretofore. On the other officers the proposition is to make a majority vote nominate. There is every reason why this should pass, as no parliamentary body in the world can succeed when a single vote may block its whole machinery.

The meeting was especially noted for a harmony of thought and interest; there were no internal dissensions, unkind words, or thoughts of any kind that could leave disappointment or heart-burnings behind. Therefore, we close, as we began, by reiterating the statement that the eighty-third annual meeting of the Medical Society of the State of New York was a memorable one.

